# Involvement of ACSL3 in the formation of autophagosomes and lipid droplets during starvation conditions

**DOI:** 10.1080/27694127.2025.2593061

**Published:** 2025-12-02

**Authors:** Shun Kato, Mitsuo Tagaya

**Affiliations:** School of Life Sciences, Tokyo University of Pharmacy and Life Sciences, Hachioji, Tokyo, Japan

**Keywords:** ACSL3, ACSL4, autophagy, lipid droplet, syntaxin 17

## Abstract

Acyl-CoA synthetase long-chain (ACSL) catalyzes the conversion of fatty acids into acyl-CoA, which is used for neutral lipid and phospholipid synthesis. Previous studies revealed that yeast Faa1 and mammalian ACSL4 play a crucial role in phagophore expansion by locally synthesizing phospholipids. We found that another member of ACSL protein family, ACSL3, which is involved in lipid droplet biogenesis under energy-rich conditions and is regulated by SYNTAXIN17, also participates in autophagosome formation, but in a different manner. Knockdown of ACSL3 suppressed punctum formation of early autophagosomal marker proteins such as FIP200 and WIPI2 in starved cells, generating nonfunctional multi-membrane autophagosome-like structures. In contrast, ACSL4 suppression blocked autophagosome formation without affecting punctum formation of early autophagosomal marker proteins. Mechanistic analysis revealed that ACSL3 functions independently of its enzymatic activity, while catalytic activity of ACSL4 is required for autophagosome formation as well as LC3 (known as MAP1LC3 proteins) protein lipidation. Furthermore, ACSL3 has been shown to be essential for lipid droplet biogenesis during starvation. These findings establish ACSL3 as a key player in two events in early autophagy: formation of autophagosomes and lipid droplets.

Acyl-CoA synthetase long-chain (ACSL) is a family of enzymes that catalyze the activation of fatty acids. Mammals possess five ACSL isoforms, ACSL1, ACSL3, ACSL4, ACSL5 and ACSL6, among which ACSL3 localizes almost uniformly to the endoplasmic reticulum (ER). Upon stimulation, it relocates to sites within the ER where lipid droplets (LDs) form, to locally supply acyl-CoA. ACSL3 depletion abrogates LD formation, suggesting a crucial role of this enzyme in LD biogenesis. Intriguingly, ACSL3 function and localization are regulated by SYNTAXIN17 (STX17), a SNARE (soluble NSF attachment protein receptor) protein implicated not only in autophagosome-lysosome fusion but also in autophagosome formation. Autophagosomes are formed de novo primarily on the ER platform through a process controlled by the autophagy-related (ATG) proteins. While it is well-known that LDs typically form under energy-rich conditions for lipid storage, their formation during nutrient starvation has also been reported, suggesting the possible activation of ACSL3 during starvation. Although the yeast homolog of ACSL4, Faa1, has been shown to contribute to autophagosome formation, the role of ACSL3 in this process remains unclear.

We investigated the involvement of ACSL3 in autophagy and compared with that of ACSL4 [1]. Knockdown of ACSL3 suppressed the degradation of p62. A flux assay using RFP-GFP-LC3 revealed that the red fluorescence puncta, indicating the presence of the reporter in autolysosomes, was not increased, although LC3 puncta were formed. ACSL3 depletion suppressed the assembly in puncta of early autophagosomal marker proteins such as FIP200 and WIPI2. In contrast, suppression of ACSL4 did not affect their punctum formation, but significantly reduced the punctum formation of LC3 proteins. Conversely, overexpression of ACSL3 and ACSL4 promoted WIPI2 and LC3 protein punctum formation, respectively. The inhibition of WIPI2 and LC3 protein punctum formation caused by suppression of ACSL3 and ACSL4 cannot be compensated by the expression of ACSL4 and ACSL3, respectively. These results suggested that ACSL3 and ACSL4 perform distinct functions in autophagosome formation (Figure 1).

**Figure 1.**
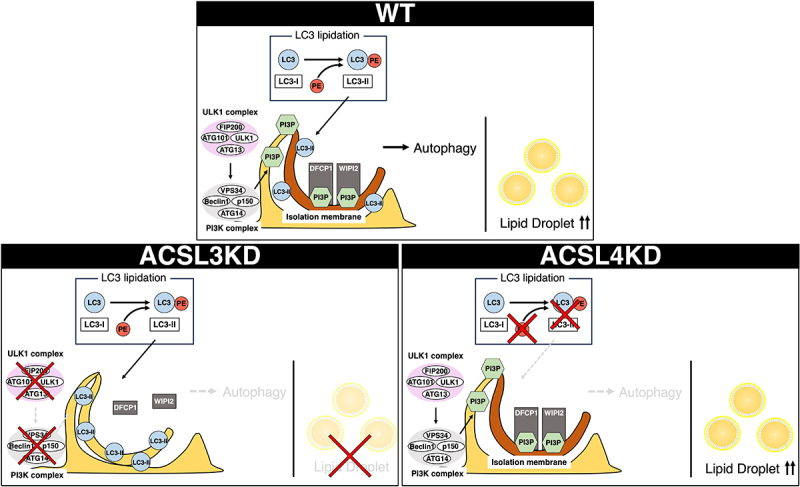
Role of ACSL3 and ACSL4 under starvation conditions. The upper panel illustrates the mechanism of autophagosome formation and the concomitant increase in lipid droplets in wild-type cells under starvation conditions. The lower left panel depicts the formation of fusion-incompetent autophagosomes and impaired lipid droplet formation in ACSL3 knockdown (KD) cells. The lower right panel shows that ACSL4 knockdown cells exhibit impaired autophagosome formation but maintain lipid droplet biogenesis.

Unexpectedly, we found that the enzymatic activity of ACSL3 is not required for WIPI2 punctum formation: WIPI2 puncta were formed in the presence of the ACSL inhibitor triacsin C and in cells expressing a catalytically-dead ACSL3 mutant. In contrast to ACSL3, LC3 protein punctum formation was inhibited by triacsin C and a catalytically-dead ACSL4 variant, confirming that the role of ACSL4 in autophagy requires its lipid synthesis activity. The finding that ethanolamine supplementation rescues the ACSL4 knockdown phenotype suggests that the enzyme-dependent function of ACSL4 is involved in LC3 protein lipidation.

One unique feature in ACSL3 knockdown cells is the formation of LC3 protein-positive structures despite the impaired recruitment of early ATG proteins. Electron microscopy revealed that these structures are not typical double-membrane autophagosomes but consist of multiple membrane layers. They failed to recruit STX17, a protein essential for autophagosome-lysosome fusion, explaining the fusion incompetency of the aberrant autophagosome-like structures. The reason for LC3 accumulation on autophagosome-like structures remains unclear, but noncanonical autophagy, which necessarily require WIPI2 for autophagosomal maturation, may be occurring.

Recent findings indicate that ACSL3 interacts with ATG9A, a key component in the early stages of autophagy. This interaction may facilitate the proper recruitment of ATG9A vesicles, to appropriate sites. ACSL3 knockdown may impair the recruitment of ATG9A, reducing the transport of other proteins via ATG9A vesicles. This may disrupt the accumulation of proper early autophagosomal marker proteins on preautophagosomal structures.

Furthermore, given that ATG9A cooperates with ATG2 proteins in mediating lipid transfer for autophagosomal membrane expansion, ACSL3 may potentially regulate ATG9A activity. Consequently, the loss of ACSL3 may lead to dysregulation of ATG9A function, resulting in defective autophagosome formation and the emergence of the multilamellar structures.

Finally, we have established that LD biogenesis during nutrient starvation is dependent on ACSL3 and STX17, as ACSL3 and STX17 knockdown inhibited this process. Furthermore, while some models have suggested that the starvation-induced increase in LDs is largely attributed to autophagy, we found that LD biogenesis occurs upon starvation even when autophagy is suppressed by ACSL4 knockdown in several cell lines. This finding may suggest that LD formation during starvation is not merely a passive product due to autophagic activity but may represent an actively induced process. In this context, LDs may be mobilized to facilitate the efficient supply of lipids for autophagosomal membrane biogenesis.

In conclusion, we show that ACSL3, in cooperation with STX17, functions as a scaffold for the formation of autophagosomes.
